# Supplementary material for: History-based action selection bias in posterior parietal cortex
Source: Nat Commun. 2017 Nov 1;8:1242. doi: 10.1038/s41467-017-01356-z (PMC5663966; doi:10.1038/s41467-017-01356-z)
Supplement: Supplementary file 1 — Supplementary Information [file 41467_2017_1356_MOESM1_ESM.pdf]

Supplementary Figures

Supplementary Figure 1

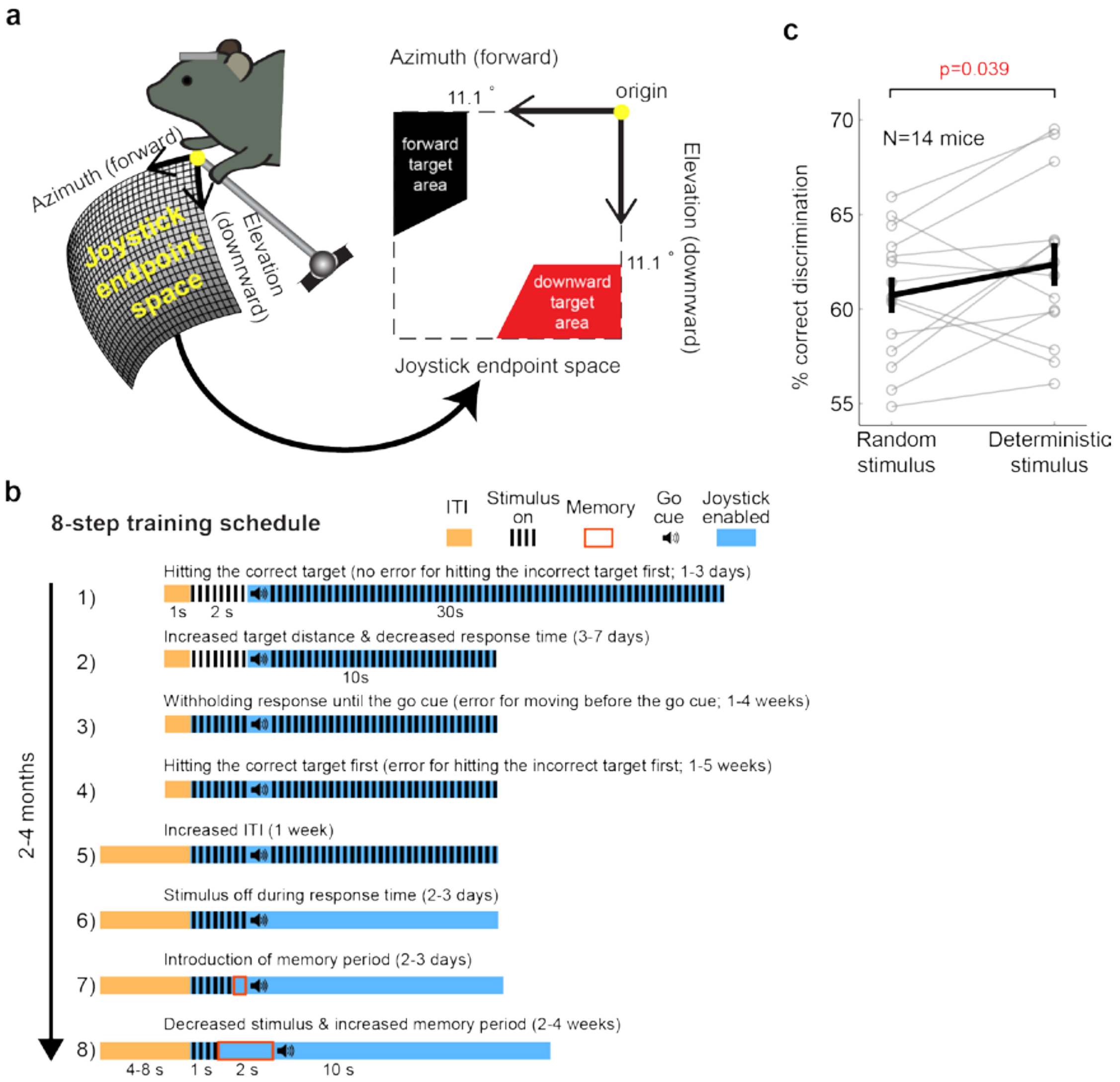

Supplementary Fig. 1. Two-alternative forced-choice tasks using the joystick apparatus.

(**a**) The two target areas in the joystick endpoint space.

(**b**) Behavioral training procedure.

(**c**) Discrimination performance in random stimulus versus deterministic stimulus trials. Stimulus was deterministic in trials following an error (the same as the previous trial) or 3 consecutive successful trials in one direction (the opposite of the previous trial), while it was random in all the other trials (Methods). Each pair of connected circles represents the average of approximately 20 sessions performed by a single mouse, and the black thick line represents mean ± s.e.m. across 14 mice. Wilcoxon one-sided signed rank test.

Supplementary Figure 2

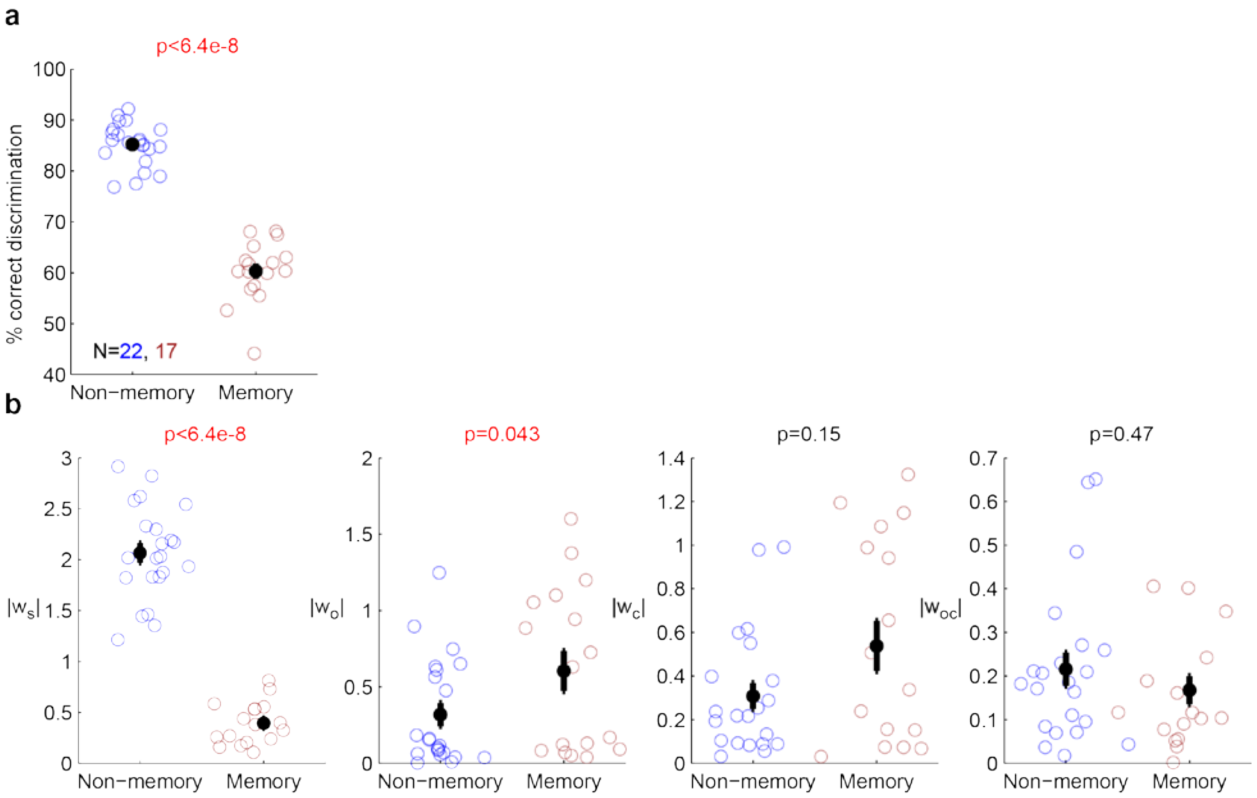

Supplementary Fig. 2. Stimulus dependency increases, while history dependency decreases in non-memory task compared to memory task.

(**a**) Discrimination performance in the non-memory (task described in step 5) and 2-sec memory trials. Each task was performed in separate sessions. Each circle represents a single session, and the black thick line represents mean ± s.e.m. Wilcoxon one-sided rank sum test.

(**b**) The magnitude of weights in the full model in the non-memory versus memory task (stimulus, outcome history, choice history, outcome-choice interaction history from left to right). The magnitude of stimulus weights are significantly larger for the non-memory than memory task. The magnitude of outcome history weight is significantly smaller for the non-memory than memory-task. Wilcoxon one-sided rank sum test.

Supplementary Figure 3

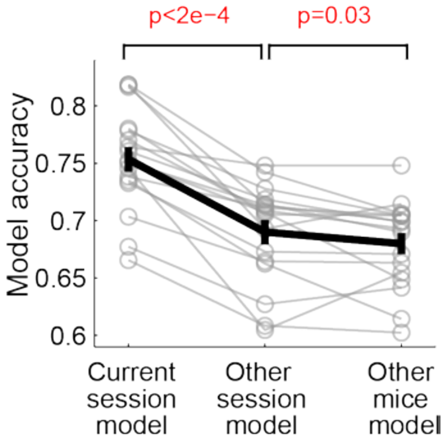

Supplementary Fig. 3. Idiosyncratic relationship between history and choice.

Choice prediction accuracy is highest when models are estimated from the current session than different sessions of the same animal, or different animals, indicating that history-dependent strategies vary across sessions and animals. Black, mean ± s.e.m. across sessions; grey, individual sessions. Wilcoxon one-sided signed rank test.

Supplementary Figure 4

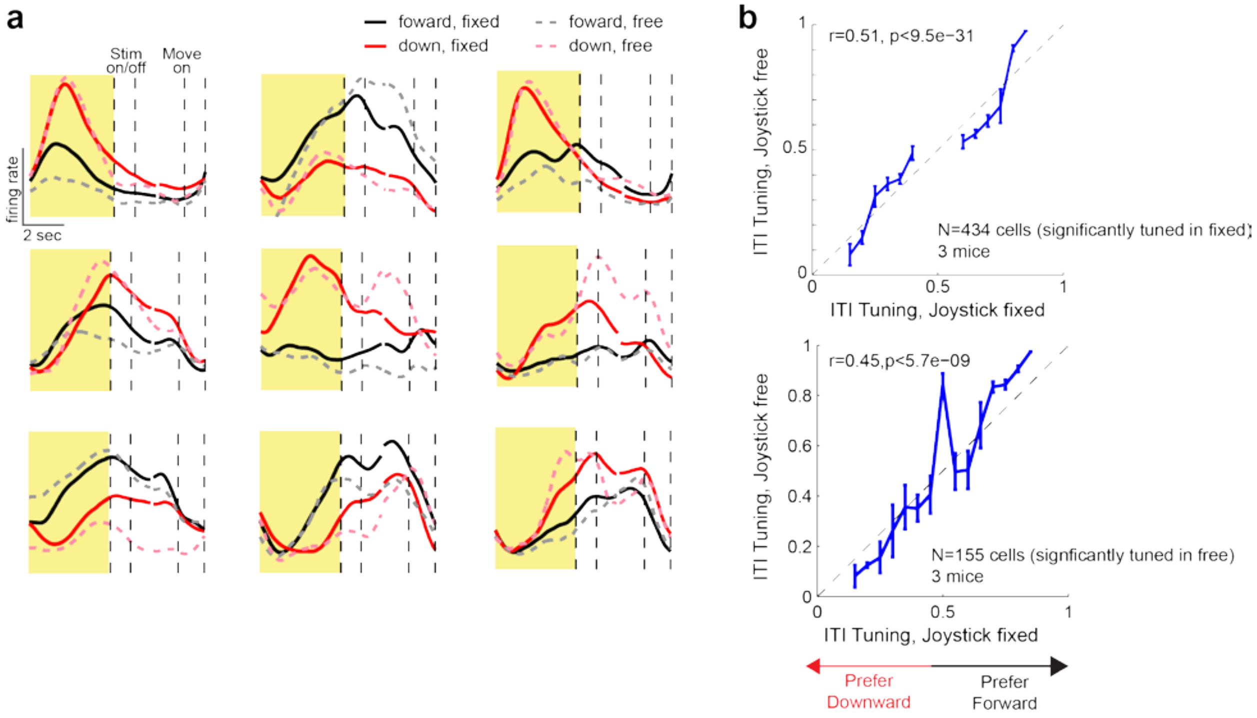

Supplementary Fig. 4. ITI choice selectivity in PPC is not a reflection of ongoing limb movements.

(**a**) Nine example neurons that are choice selective during the ITI (yellow shade) in the original condition in which the joystick was fixed during the ITI. The average activity for forward (black) versus downward (red) choice trials in the fixed-joystick (solid) and free-joystick conditions (dashed). Fixed- and free-joystick conditions were randomly interleaved, and approximately 15% were free-joystick trials. The four dotted vertical lines mark stimulus onset, offset, movement onset, and the next trial ITI onset.

(**b**) The choice tuning strength during the ITI in the fixed- versus free-joystick condition for 434 cells that are significantly choice selective during the ITI in the fixed-joystick condition (Mean ± s.e.m.). The black dashed line represents the unity line.

(**c**) The same as b, but for 155 cells that are significantly choice selective during the ITI in the free-joystick condition (Mean ± s.e.m.). The black dashed line represents the unity line.

Supplementary Figure 5

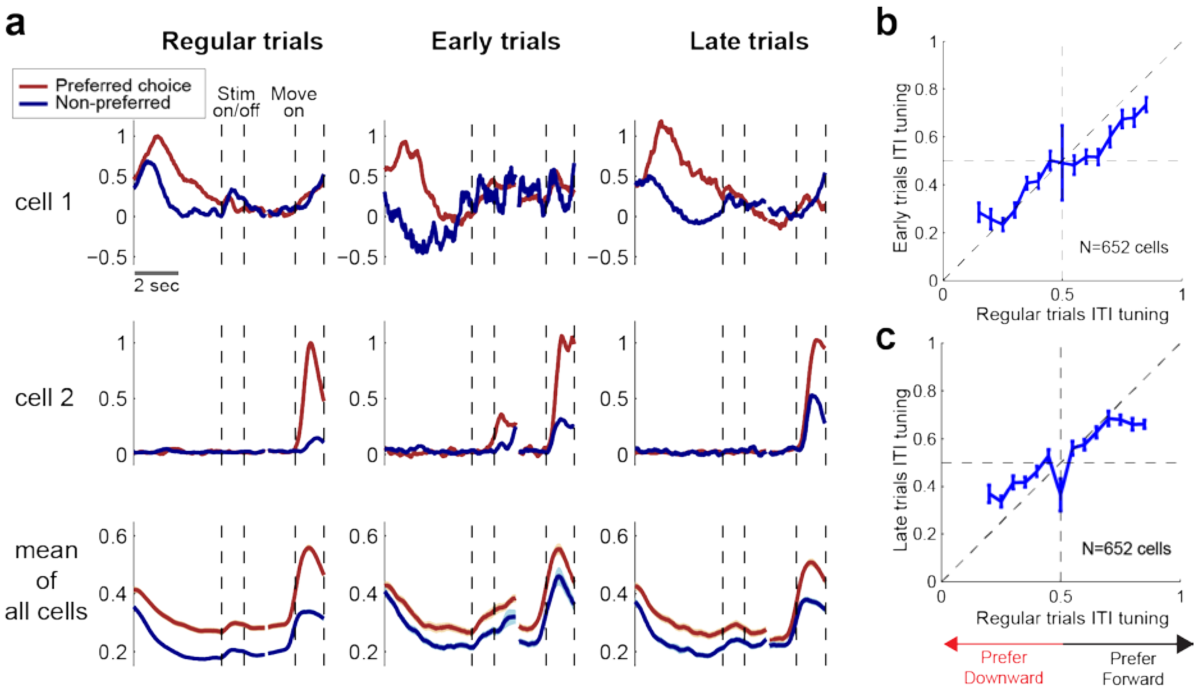

Supplementary Fig. 5. ITI choice tuning persists in early and late execution trials.

(**a**) The activity in preferred (red) versus non-preferred (blue) trials for example cells 1-2, and the mean activity of all choice cells in regular, early, and late trials.

(**b**) ITI tuning of regular versus early trials (Mean ± s.e.m.). The dashed line represents the unity line.

(**c**) ITI tuning of regular versus late trials.

Supplementary Figure 6

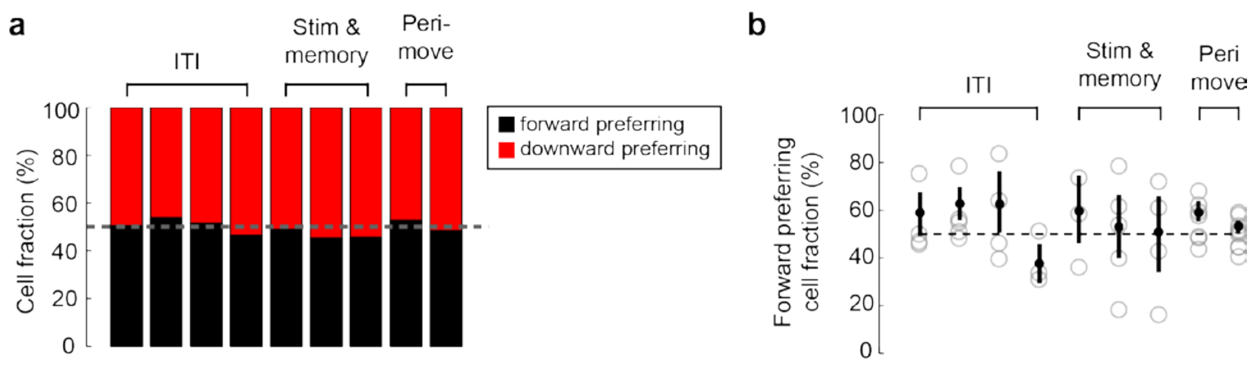

Supplementary Fig. 6. The preferred directions of choice selectivity are equally distributed.

(**a**) Fractions of cells preferring forward versus downward choice across all choice-selective PPC cells in 11 non-overlapping 1-sec epochs.

(**b**) Fraction of cells preferring forward choice in individual imaging fields with at least 15 choice-selective cells in a given epoch. Dots are individual fields and the thick black line indicates mean ± s.e.m.

Supplementary Figure 7

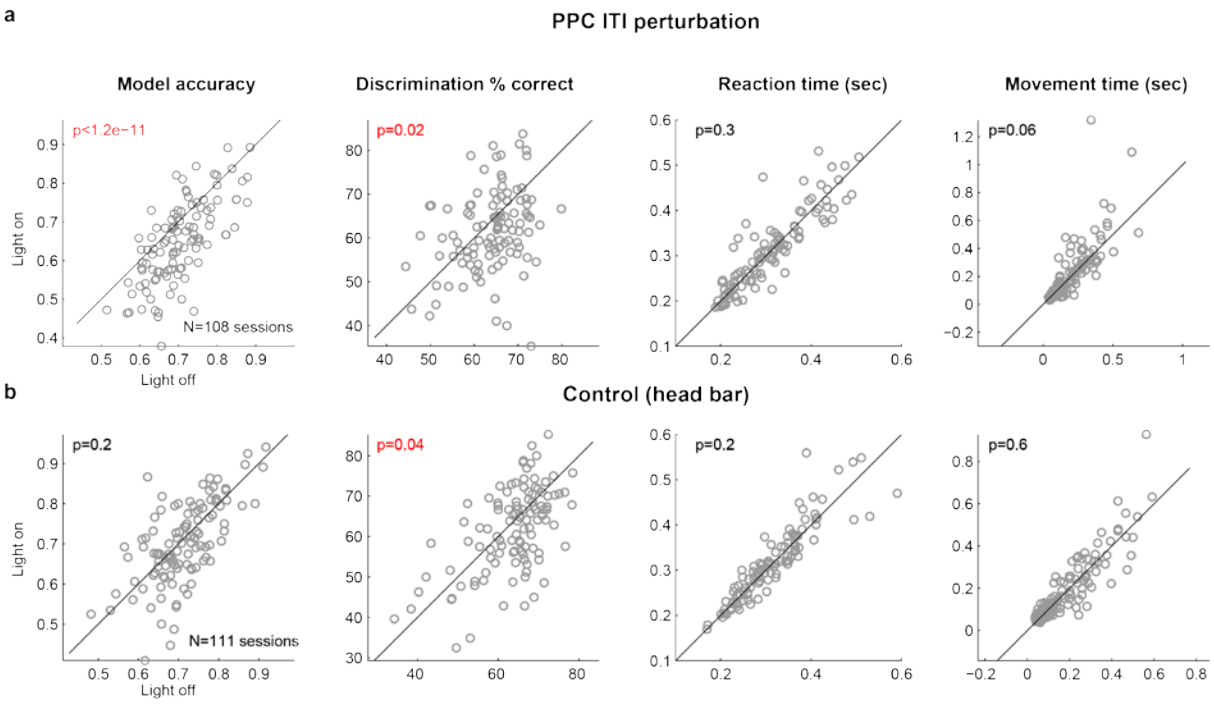

Supplementary Fig. 7. PPC ITI inactivation effect on internal model accuracy, task performance, reaction time, and movement time.

(**a**) Inactivation sessions. Each circle represents a single session. The x-axis is the mean of light-off trials in a given session, and the y-axis is the mean of light-on trials. The black lines represent the unity line. Wilcoxon signed rank test for difference between the light-off and light-on trials.

(**b**) Control sessions.

Supplementary Figure 8

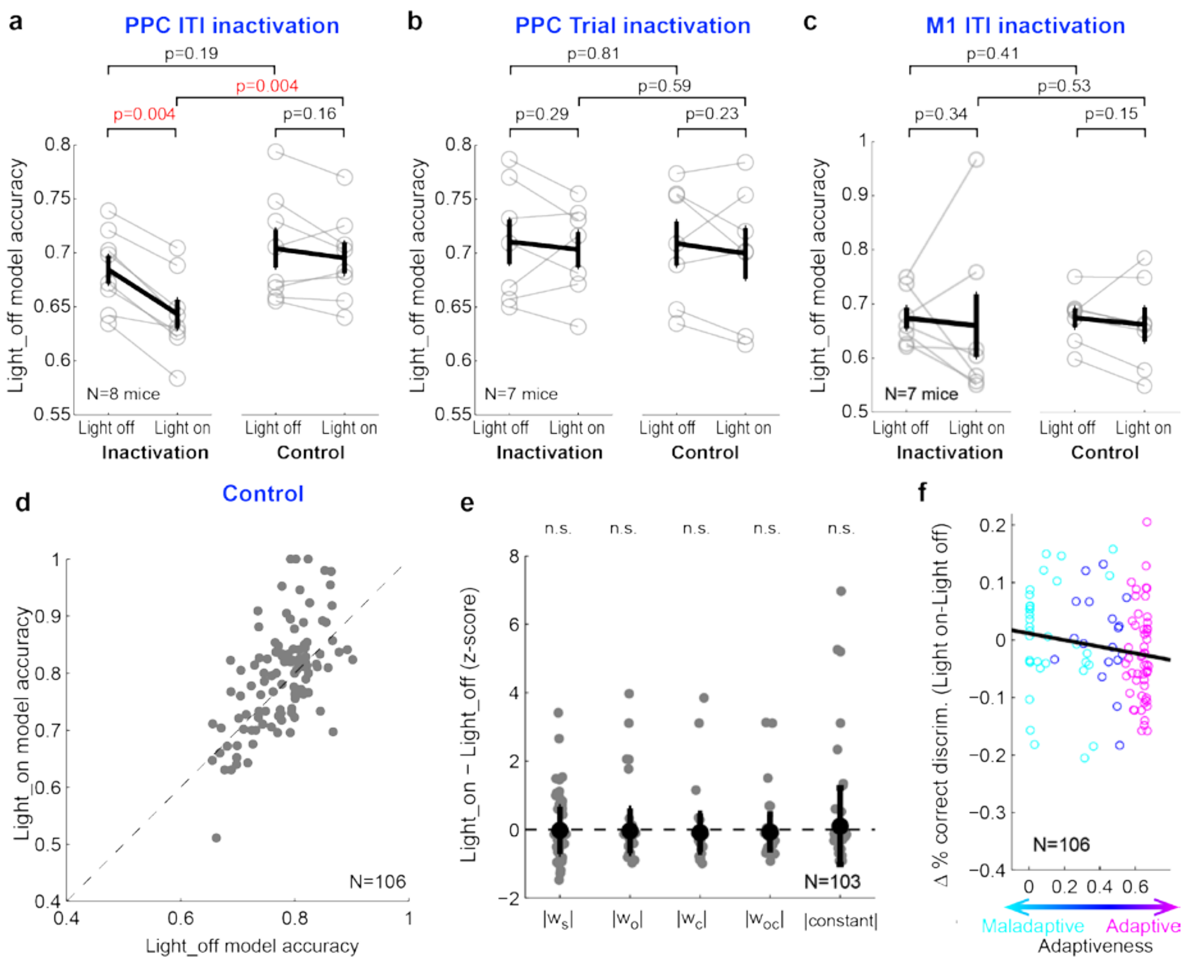

Supplementary Fig. 8. Inactivation control sessions.

(**a-c**) Average light-off model accuracy in light-off and light-on trials in control sessions. Black, mean ± s.e.m. across mice; colors, individual mice. Wilcoxon one-sided signed rank test. Compare these to **Fig. 7d**,**f**,and **g**. There is no significant non-specific effects of blue light.

(**d**) The light-on model accuracy versus light-off model accuracy in control sessions. PPC ITI and M1 ITI control sessions are combined (N=106). The light-on model accuracy is not significantly different from the mean light-off model accuracy in control sessions (Wilcoxon signed rank test, p=0.08). Compare this plot to **Fig. 8a**.

(**e**) Changes in the magnitude of model weights in ITI control sessions. No weight shows significant change by blue light (bootstrap, p>0.05). In the sessions with a model accuracy of 1 (N=3), animals made choice in only one direction in light-on trials. These sessions were removed from the analysis because of unreliable weight estimates (see Supplemental Information). Compare this plot to **Fig. 8b**.

(**f**) Change in discrimination performance by blue light during ITI (light-on – light-off) as a function of the degree of adaptiveness of the strategies (cyan: maladaptive, blue: neutral, and magenta: adaptive strategy sessions, respectively). Compare this plot to **Fig. 8c**.
